# The association between premorbid beta blocker exposure and mortality in sepsis—a systematic review

**DOI:** 10.1186/s13054-019-2562-y

**Published:** 2019-09-04

**Authors:** Kaiquan Tan, Martin Harazim, Benjamin Tang, Anthony Mclean, Marek Nalos

**Affiliations:** 1 Nepean Clinical School, Sydney Medical School, University of Sydney, Penrith, Australia; 2 Department of Intensive Care Medicine, Nepean Hospital, Penrith, Australia; 3 Centre for Immunology and Allergy Research, Westmead Millennium Institute, Westmead, Australia; 4 Medical Intensive Care Unit, Teaching Hospital and Biomedical Centre, Charles University, Alej Svobody 80, 323 00 Pilsen, Czech Republic

**Keywords:** Sepsis, Mortality, Beta blockers, Systematic review

## Abstract

**Background:**

The effect of premorbid β-blocker exposure on clinical outcomes in patients with sepsis is not well characterized. We aimed to examine the association between premorbid β-blocker exposure and mortality in sepsis.

**Methods:**

EMBase, MEDLINE, and Cochrane databases were searched for all studies of premorbid β-blocker and sepsis. The search was last updated on 22 June 2019. Two reviewers independently assessed, selected, and abstracted data from studies reporting chronic β-blocker use prior to sepsis and mortality. Main data extracted were premorbid β-blocker exposure, mortality, study design, and patient data. Two reviewers independently assessed the risk of bias and quality of evidence.

**Results:**

In total, nine studies comprising 56,414 patients with sepsis including 6576 patients with premorbid exposure to β-blockers were eligible. For the primary outcome of mortality, two retrospective studies reported adjusted odds ratios showing a reduction in mortality with premorbid β-blocker exposure. One study showed that premorbid β-blocker exposure decreases mortality in patients with septic shock. Another study showed that continued β-blockade during sepsis is associated with decreased mortality.

**Conclusion:**

This systematic review suggests that β-blocker exposure prior to sepsis is associated with reduced mortality. There was insufficient data to conduct a bona fide meta-analysis. Whether the apparent reduction in mortality may be attributed to the mitigation of catecholamine excess is unclear.

**Trial registration:**

PROSPERO, CRD42019130558 registered June 12, 2019.

**Electronic supplementary material:**

The online version of this article (10.1186/s13054-019-2562-y) contains supplementary material, which is available to authorized users.

## Introduction

The Sepsis-3 consensus defines sepsis as a life-threatening organ dysfunction caused by a dysregulated host response to infection [1]. While our understanding of sepsis pathophysiology is increasing, controversies in haemodynamic management persist [2, 3]. The most recent surviving sepsis guidelines recommend noradrenaline as the first-choice vasopressor because of its vasopressor and positive inotropic properties [strong recommendation, moderate quality of evidence [4]]. In contrast, the concept of ‘decatecholamisation’ emerged in the last decade stemming from the recognized negative effects of catecholamines in sepsis [3, 5, 6]. Interestingly, the β-adrenergic blockade has emerged as a possible treatment option for blunting the adrenergic response in early sepsis with potential effects on the modulation of cardiogenic, metabolic, immunologic, and coagulopathic derangements in sepsis [7].

Early administration of the short-acting β-blocker esmolol in a recent trial showed a reduction in 28-day sepsis mortality [8, 9]. Furthermore, some studies have suggested a benefit of premorbid β-blocker exposure on sepsis outcomes [10, 11]. Multiple systematic reviews have since concluded that there is limited preliminary evidence for the use of β-blockers during sepsis [12–14], while others are skeptical [15]. However, to date, no published systematic review exists on the effects of premorbid β-blocker exposure on sepsis outcomes, including mortality. Therefore, we set out to systematically examine the evidence from all human studies on premorbid β-blocker exposure and sepsis.

## Materials and methods

This study follows the Meta-analysis Of Observational Studies in Epidemiology (MOOSE) guidelines [16] and was registered with the international prospective register of systematic reviews (PROSPERO; CRD 42019130558). The MOOSE checklist is appended as Additional file 1: Table S1.

### Data sources and searches

Three databases, EMBase, MEDLINE, and Cochrane were searched on 30 January 2019 for records dating from database conception to the date of search that was last updated on 22 June 2019. The search was only limited to human research. Duplicates were removed using the Ovid platform and checked for any incorrect removal. Hand searching from reference lists was also performed. The full search strategy is appended as Additional file 4: Figure S1.

### Study selection

Inclusion criteria for this review were guided by the ‘Patient, Population, or Problem, Intervention, Comparison, Outcome, Study Design or Setting’ (PICOS) framework [17] (Table 1). Patients exposed to β-blockers prior to an episode of sepsis or septic shock and were cared for in the emergency department (ED) or intensive care unit (ICU) were included in this review. Observational studies were eligible. Excluded were case studies/small series (< 20 patients overall) and review articles. The abstracts were assessed by two investigators (KT, MH) independently, and disagreements were resolved with a third investigator (MN).

**Table 1:** ‘PICOS’ approach for selecting clinical studies in the systematic search. PICOS Patient, Population, or Problem, Intervention, Comparison, Outcome, Study Design or Setting

PICOS	Study characteristics
1. Participants	Patients with sepsis and/or septic shock
2. Intervention	Premorbid exposure to beta blockers
3. Comparison	No premorbid exposure to beta blockers
4. Outcomes	Mortality
5. Study design	Prospective observational or retrospective cohort studies

### Data extraction and quality assessment

Data from eligible studies were independently extracted by two investigators (KT, MH). Where required, study authors were contacted directly to kindly provide missing research data. The Risk Of Bias In Non-randomized Studies - of Interventions (ROBINS-I) tool [18] was used to independently assess (KQ, MH) the quality of studies.

### Data synthesis and analysis

Adjusted outcome data were combined using the inverse variance method [19]. Heterogeneity between studies was measured by Higgin’s and Thomson’s *I*^2^ [20]. Statistical analyses were performed using Review Manager version 5.3 (Copenhagen: The Cochrane Collaboration, 2014)

## Results

### Study selection

**Fig. 1:**
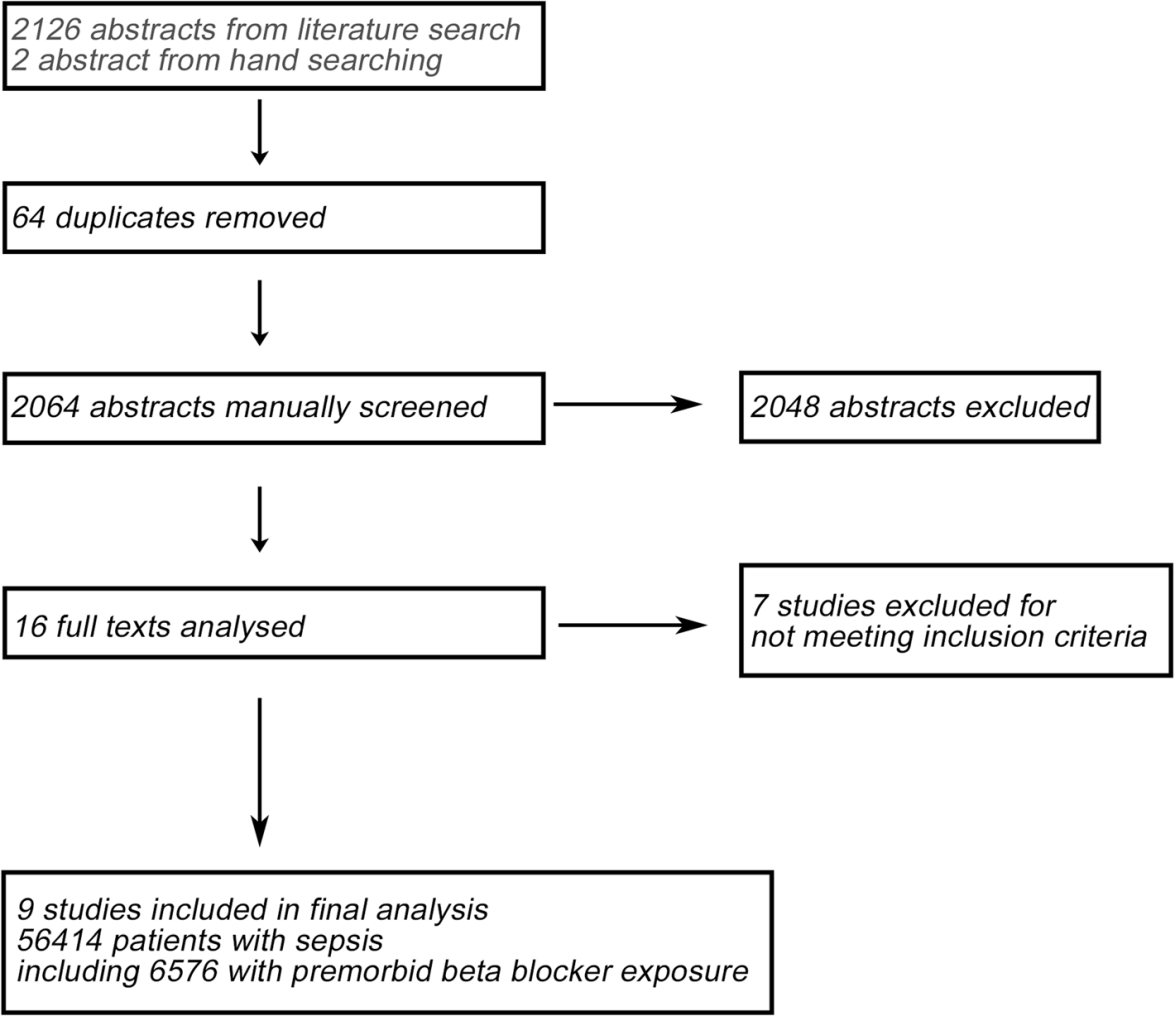
Flow diagram of the study selection process

The initial search returned 2128 abstracts, all in English. Two thousand sixty-four abstracts were manually screened after removal of 64 duplicates. After screening, 16 studies were initially selected for data extraction. Where required, the corresponding authors were contacted to obtain necessary data for statistical analysis. Seven studies were excluded for not meeting all inclusion criteria. The list of studies excluded is appended (Additional file 2: Table S2). Overall, a total of nine studies were found to be eligible, comprising 56,414 patients with sepsis, including 6576 patients with premorbid exposure to β-blockers (Fig. 1).

### Characteristics and type of studies

All studies were retrospective cohort studies, and the data were collected between 1999 and 2017.

The study populations described patients with sepsis, severe sepsis, or septic shock in an ED [21] or ICU [10, 11, 21–26] setting. The definitions of sepsis, severe sepsis, septic shock, and premorbid β-blocker exposure varied slightly across the studies, but were reasonable and comparable to current definitions [1]. Two studies [10, 27] included patients with sepsis, severe sepsis, and septic shock, while seven studies [11, 21–26] included patients with severe sepsis and/or septic shock. One study (Alsolamy et al.) included patients ≥ 14 years of age; all other studies included adult (18 years and above) patients. Four studies by Sharma et al., Charles et al., Alsolamy et al., and Al-Qadi et al. were reported as conference abstracts [22–25]. The characteristics of the studies are appended (Table 2).

**Table 2:** Characteristics of included studies

First author	Year of publication	Type of study	Study period (month/year)	Country	Number of centres	Diagnosis	Setting (ED/ICU)	ICU type (medical/surgical)	Outcome	Premorbid beta blocker exposure	Inclusions	Exclusions	Select cohort	No. of patients with premorbid beta blocker use
Singer et al. [11]	2017	Retrospective matched cohort study	2009–2011	USA	Medicare Provider and Analysis Review data	Severe sepsis, septic shock	ICU	Mixed	Primary: mortality	Filled prescription within 30 days of admission, including date of admission. Data obtained from Medicare Part D claims data.	Patients admitted with an urgent/emergent hospital admission code, requiring intensive care upon admission, and carrying a primary diagnosis of sepsis or systemic inflammatory response syndrome (SIRS) by ICD9 diagnosis codes, > 65 years, asthma, heart block, 1 year of continuous Part A and Part B coverage, with Part D enrolment.	In hospital claims without ICU admission, Part C enrolment (coverage through healthcare maintenance organizations), beta blocker prescriptions extending into 30 days prior to admission but not through the admission date.	6839	2838
Macchia et al. [10]	2012	Retrospective matched cohort study	2003–2008	Italy	22	Sepsis	ICU	Mixed	Primary: mortality	3 or more filled prescriptions within 4 months of admission. Data obtained from local health authority drug claims database.	Sepsis with codes 038 [septicemia], 020.0 [septicemic], 790.7 [bacteremia], 117.9 [disseminated fungal infection], 112.5 [disseminated candida infection], and 112.81 [disseminated fungal endocarditis]. Admission direct to ICU or transferred to ICU from other hospital departments within 48 h of admission.	< 40 years old, transfer to ICU from other departments 48 h after admission.	9465	1061
Hsieh et al. [27]	2019	Retrospective matched observational study	1999–2013	Taiwan	National Health Insurance Research Database (NHIRD) of Taiwan data	Sepsis, septic shock	–	–	Primary: mortality	Patients were classified as using certain drugs if they took them for more than 1 week within a 3-month period prior to the index date.	First episode of severe sepsis or septic shock. ICD-9-CM coding was used.	Second episode of sepsis.	33,213	1040
Fuchs et al. [26]	2017	Secondary analysis of prospective observational single-centre trial	2010–2013	Germany	1	Sepsis, severe sepsis, septic shock	ICU	Surgical	Primary: mortality; survival analysis Secondary: length of stay	Pre-existing oral beta blocker therapy was defined as a treatment started at least 7 days before sepsis onset.	First episode of severe sepsis or septic shock.	< 18 years old, no infection, no chronic beta blocker therapy, no sepsis or septic shock, second episode of sepsis.	296	296
Contenti et al. [21]	2015	Retrospective cohort study	2012–2014	France	1	Severe sepsis, septic shock	ED/ICU	–	Primary: initial blood lactate concentration. Secondary: mortality	‘Premorbid’ not defined. Data obtained from ED observation data or inpatient records	> 18 years old, severe sepsis, septic shock.	–	260	65
Sharma et al. [25]	2016	Retrospective study	2013–2014	ICU	Not specified	Septic shock	ICU	Medical	Peak lactate, cumulative norepinephrine dose and duration, mortality	‘Premorbid’ not defined. Data obtained from ICU medical records.	Medical ICU, septic shock, vasopressors required.	–	123	48
Charles et al. [23]	2018	Retrospective study	2008–2016	France	Not specified	Septic shock	ICU	–	Heart rate, arterial lactate levels, arterial oxygen content, fluid requirements, norepinephrine requirements, duration of mechanical ventilation, mortality	‘Premorbid’ not defined.	Adult patients diagnosed with septic shock within 48 h.	–	938	230
Alsolamy et al. [22]	2016	Retrospective cohort study	1/1/2003–31/12/2013	Saudi Arabia	1	Severe sepsis, septic shock	ICU	–	Primary: mortality	Active prescription 3 months prior to admission.	> 14 years old, severe sepsis and septic shock, previous prescription of beta blockers active for 3 months prior to hospital admission.	–	4629	623
Al-Qadi et al. [24]	2014	Retrospective study	2007–2009	USA	1	Severe sepsis, septic shock	ICU	Medical	Primary: mortality	3 or more months of beta blocker usage prior to admission. Data obtained from electronic records.	Severe sepsis and septic shock, 3 or more months on beta blocker prior to ICU admission.	Patients with comfort care.	651	375

### Risk of bias assessment

All observational studies of premorbid medication use are at risk of bias because of confounding. Five studies included in this review [10, 11, 24, 26, 27] were judged to be of moderate risk of bias for the primary outcome of mortality as they reported adjustment of confounding variables via statistical analysis. Four studies [21–23, 25] were judged as having serious risk of bias due to confounding as the authors did not perform statistical analysis to correct for confounders. The risk of bias assessment using ROBINS-1 tool for each trial is appended (Additional file 5: Figure S2) with reasonings attached (Additional file 3: Table S3).

### Primary outcome: mortality

The smallest study by Contenti et al. included 260 sepsis patients. Results from that study showed a non-significant decrease in 28-day mortality (35% vs 49%, *p* = 0.08; Table 3). Using multivariate logistic regression, three studies by Singer et al., Macchia et al., and Hsieh et al. reported mortality data as adjusted odds ratios [10, 11, 27]. Singer et al. reported a decrease in hospital mortality in patients with severe sepsis and septic shock (aOR = 0.69; 95% CI [0.62, 0.77]; Table 3). Subgroup analysis between cardioselective β-blockers and non-selective β-blockers showed that non-selective β-blockers were associated with lower hospital mortality, adjusted OR for non-selective β-blockers (aOR = 0.59; 95% CI [0.49, 0.71]) compared to cardioselective β-blockers (aOR = 0.73; 95% CI [0.65, 0.82]). Overall mortality rate for cardioselective β-blocker users was higher, cardioselective β-blocker users vs. non-selective β-blocker users (aOR = 1.23; 95% CI [1.11–1.36]). Hospital mortality was also reduced across all age groups: between ages 65 and 74 (aOR = 0.64; 95% CI [0.52, 0.80]), between ages 75 and 84 (aOR = 0.69; 95% CI [0.58, 0.83]), and above 85 (aOR = 0.73; 95% CI [0.60, 0.90]).

**Table 3:** Mortality data for included studies. Premorbid beta blocker exposure vs no premorbid beta blocker exposure

First author	Select cohort	No. of patients with no premorbid beta blocker use	No. of patients with premorbid beta blocker use	Mortality census day	Mortality	90-day mortality	28-day mortality	ICU mortality	Hospital mortality	Survival analysis	Outcome	Adjustment method	Adjusted variables
Singer et al. [11]	6839	4001	2838	Hospital mortality	–	–	–	–	aOR = 0.69 (CI 0.62–0.77)	–	Premorbid beta blocker usage is significantly associated with decreased mortality	Multivariate logistic regression	Age, class of beta blocker, congestive heart failure, cancer, surgical procedures
Macchia et al. [10]	9465	8404	1061	28-day mortality	–	–	aOR = 0.81 (CI 0.68–0.97), *p* = 0.025	–	–	–	Premorbid beta blocker usage is significantly associated with decreased mortality	Multivariate logistic regression	Age, sex, history of hypertension, dyslipidaemia, diabetes mellitus, myocardial infarction, congestive heart failure, atrial fibrillation, chronic obstructive pulmonary disease, depression, and malignancy
Hsieh et al. [27]	33,213	32,173	1040	Hospital mortality	–	–	–	–	aOR = 0.89 (CI 0.76–1.04), *p* = 0.1484	–	Premorbid beta blocker usage is not significantly associated with decreased mortality	Multivariate logistic regression	Age, sex, insurance premium, urbanization level, and comorbidities
Fuchs et al.^a^ [26]	296	0	296	ICU, hospital, 28 days, 90 days	–	40.7% vs. 52.7%, *p* = 0.046^a^	28.7% vs. 41.1%, *p* = 0.04^a^	27.5% vs. 38%, *p* = 0.06^a^	35.3% vs. 48.1%, *p* = 0.03^a^	HR = 0.67 (CI 0.48, 0.95), *p* = 0.03^a^	Continuation of beta-blockade is associated with decreased 28-day, 90-day, and hospital mortality.	Multivariate cox regression	Sex, known nosocomial pathogen, chronic diseases, body temperature (< 36.0 °C), APACHE II score first 24 h, lactate first 24 h (> 3 mmol/L)
Contenti et al. [21]	260	195	65	28-day mortality	–	–	–	–	35% vs 49%, *p* = 0.08	–	Premorbid beta blocker usage is not significantly associated with decreased mortality	–	–
Sharma et al. [25]	123	75	48	Hospital mortality	–	–	–	–	35.4% vs 32%, *p* = 0.70	–	Premorbid beta blocker usage is not significantly associated with decreased mortality	–	–
Charles et al. [23]	938	708	230	ICU mortality	–	–	–	35.7% vs. 37%, *p* = 0.75	–	–	Premorbid beta blocker usage is not significantly associated with decreased mortality	–	–
Alsolamy et al. [22]	4629	4006	623	ICU mortality	–	–	–	RR = 0.94 (CI 0.82–1.08), *p* = 0.39	–	–	Premorbid beta blocker usage is not significantly associated with decreased mortality	–	–
Al-Qadi et al. [24]	651	276	375	Not specified	21.3% vs 27.2%, *p* = 0.09; aOR 0.62, *p* = 0.023	–	–	–	–	–	Premorbid beta blocker usage is not significantly associated with decreased mortality	–	Age, gender, and severity of illness using SOFA and APACHE III scores

^a^Continued beta blocker usage during sepsis vs discontinued beta blocker usage during sepsis

Macchia et al. reported a significant decrease in 28-day mortality in patients with sepsis (aOR = 0.81; 95% CI [0.68–0.97]; *p* = 0.025; Table 3). Subgroup analysis investigating the effect of age, gender, organ dysfunction, and previous comorbidities did not alter the results. Adjustment for previous medication used including calcium channel blockers, amiodarone, angiotensin-converting-enzyme inhibitors, diuretics, or any nonsteroidal anti-inflammatory drugs also did not alter the results. The authors also conducted a propensity matching analysis, which led to similar results (OR = 0.72; 95% CI [0.57–0.91]; *p* = 0.04).

The study by Hsieh et al. showed that premorbid β-blocker exposure was not associated with a significant decrease in hospital mortality in patients with sepsis and septic shock (aOR = 0.89; 95% CI [0.76, 1.04]; *p* = 0.1484; Table 3). However, subgroup analysis of patients with septic shock showed that premorbid β-blocker exposure was significantly associated with decreased hospital mortality (aOR = 0.68; 05% CI [0.56, 0.82]; *p* = 0.0001). In patients without septic shock, premorbid β-blocker exposure was associated with significantly higher mortality (aOR = 1.16; 95% CI [1.11, 1.21]; *p* < 0.0001).

**Fig. 2:**
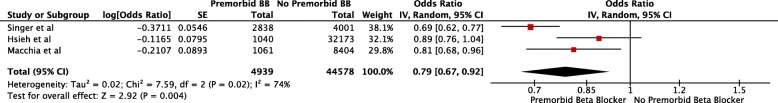
Adjusted odds ratio analysis via forest plot of sepsis mortality rates in studies comparing populations with premorbid β-blocker (BB) exposure to populations without premorbid β-blocker exposure. Horizontal bars represent 95% confidence intervals

We compared the mortality data from the three studies that adjusted for potential confounders. Pooled analysis of the three studies showed an average odds ratio, aOR = 0.79; 95% CI (0.67, 0.92), *p* = 0.004; Fig. 2. However, there was substantial heterogeneity (*i*^2^ = 74%) between the studies, indicating that a meta-analysis is premature and that further studies and subgroup analyses are needed to validate the results.

Our systematic search also included grey literature in the form of conference abstracts. Mortality data from three studies reported as conference abstracts showed a trend towards a decrease in mortality with premorbid β-blocker exposure. However, the results were not statistically significant: Charles et al. (ICU mortality; 35.7% vs. 37%, *p* = 0.75), Alsolamy et al. (ICU mortality; RR = 0.94 (CI: 0.82–1.08), *p* = 0.39), and Al-Qadi et al. (21.3% vs 27.2%, *p* = 0.09) (Table 3). Of note, the study by Alsolamy et al. included patients ≥ 14 years old, while all other studies only included adults. Another retrospective study, reported as a conference abstract, involving 123 sepsis patients showed a non-significant increase in mortality with premorbid β-blocker exposure: Sharma et al. (hospital mortality; 35.4% vs 32%, *p* = 0.70; Table 3).

One interesting study by Fuchs et al. investigated the effect of continuing premorbid β-blocker use in patients with severe sepsis and septic shock. This study included 296 patients on chronic β-blockers, in which β-blockade was continued in 176 patients. Results showed that continuation of β-blockade during sepsis was associated with decreased 28-day (28.7% vs. 41.1%, *p* = 0.04), 90-day (40.7% vs. 52.7%, *p* = 0.046), and hospital mortality (35.3% vs. 48.1%, *p* = 0.03) (Table 3). Survival analysis also indicated that continuation of β-blockade during sepsis is significantly associated with decreased mortality (HR = 0.67; 95% CI [0.48, 0.95]; *p* = 0.03; Table 3).

### Clinical parameters

Only four studies by Contenti et al. [21], Charles et al. [23], Sharma et al. [25], and Fuchs et al. [26] provided clinical parameter data. However, reporting of parameters was inconsistent. There was no significant difference in the requirements for vasopressor infusion across all four studies. Contenti et al. and Charles et al. found that premorbid β-blocker exposure was associated with decreased heart rate; Sharma et al., did not report heart rate data. Continuation of β-blockade during sepsis was not associated with a decrease in heart rate in the first 24 h [26]. Premorbid β-blocker use was found to be associated with lower initial plasma lactate levels by Contenti et al., but not by Charles et al.. The continuation of β-blockade during sepsis was associated with lower plasma lactate levels in the first 24 h [26].

There were no significant differences in all other relevant parameters including mean arterial pressure, Sequential Organ Failure Assessment (SOFA) score, Acute Physiology and Chronic Health Evaluation (APACHE)-II or III score, and incidence of mechanical ventilation. The clinical parameter data are presented in Table 4.

**Table 4:** Reported clinical parameters

First author	Heart rate	Initial blood lactate levels	Peak blood lactate levels	Creatinine levels	Arterial pH	Mean arterial pressure	SOFA score	APACHE II score	APACHE III score	Mechanical ventilation	Vasopressor infusion
Contenti et al.^a^ [21]	100 ± 25 vs 109 ± 25 bpm; *p* = 0.02	3.9 ± 2.3 mmol/L vs 5.6 ± 3.6 mmol/L; *p* = 0.0006	–	–	–	72 mmHg ± 22 vs 70 mmHg ± 21; *p* = 0.48	5.0 ± 2.8 vs 5.3 ± 2.8; *p* = 0.44	21.0 ± 6.0 vs 21.7 ± 6.9; *p* = 0.41	–	15% vs 19%; *p* = 0.58	31% vs 32%; *p* = 0.94
Sharma et al.^a^ [25]	–	–	3.2 vs 3.6 mmol/L; *p* = 0.54	–	–	–	–	–	94 vs 84; *p* = 0.14	–	Cumulative dose 11.4 vs 12.6 mg; *p* = 0.43 Duration of infusion 1563 vs 1730 min; *p* = 0.37
Charles et al.^a^ [23]	81 (IQR 82–111) vs. 107 (IQR 89–122) bpm; *p* < 0.01	1.75 (IQR 0.9–3.4) vs. 1.8(IQR 0.8–4) mmol/L; *p* = 0.97	–	165.5 (IQR 108–245) vs 135.5 (IQR 82–108); *p* < 0.00	7.35 (IQR 7.25–7.42) vs 7.34 (IQR 7.23–7.42); *p* = 0.354	–	9 (IQR 6–12) vs 9 (IQR 6–13); *p* = 0.242	–	–	81.7% vs 84.9%; *p* = 0.112 Days on ventilation 4 (IQR 2–9) vs 5.5 (IQR 2–11); *p* = 0.055	23.2 mg (IQR 5.1–57.0) vs 22.4 mg (IQR 5.2–60.5); *p* = 0.95
Fuchs et al.^b^ [26]	111 (IQR 97.0–132.8) vs 118 (IQR 97.0–135.5); *p* = 0.2	2.3 (IQR 1.5–3.8) vs 3.5 (IQR 2.0–6.5); *p* < 0.01	–	–	–	–	–	20.0 (IQR 15.0–24.5) vs 21.0 (IQR 16.2–26.0); *p* = 0.25	–	–	Norepinephrine 91% vs 92.2%; *p* = 0.83

^a^Premorbid beta blocker exposure vs no premorbid beta blocker exposure

^b^Continued beta blocker usage during sepsis vs discontinued beta blocker usage during sepsis

## Discussion

This is the first systematic review examining the role of premorbid β-blocker exposure on mortality outcomes in patients with sepsis. While there was not enough data to conduct a meta-analysis, pooled adjusted odds ratio from three studies indicated a potential decrease in mortality associated with premorbid β-blocker use, albeit with substantial heterogeneity. Our results provide preliminary evidence of a potential association between premorbid β-blocker use and mortality in sepsis and add to the emerging evidence suggesting harmful effects of adrenergic stress on mortality in sepsis. We discuss the effects of premorbid β-blocker exposure on the adrenergic response in early sepsis.

Cardiac dysfunction in sepsis is common and has both systolic and diastolic components [5]. However, only diastolic dysfunction seems to be associated with mortality [28, 29]. While being on premorbid β-blockers may reduce systolic function, the reduction of adrenergic response in sepsis (decreasing heart rate, prolongation of diastolic time, and improved coronary perfusion) can lead to mitigation of diastolic dysfunction [28, 29]. Further, the risks of myocardial ischemia may be decreased due to reduced myocardial oxygen consumption [14].

Patients with septic shock are often treated with large doses of exogenous catecholamines for haemodynamic stabilization. The most recent Surviving Sepsis Campaign guidelines recommend using noradrenaline as the first-line agent for vasopressor therapy, with adrenaline or low-dose vasopressin as second-line agents [4]. Increased dosage and duration of noradrenaline administration has been associated with higher incidence of new onset atrial fibrillation [3]. Excessive catecholamine levels may also play an important role in sepsis-related cardiac dysfunction by causing cardiomyopathy and cardiomyocyte necrosis [5, 7]. β-adrenergic blockade could reduce the amount of exogenous catecholamines used by restoring sepsis-induced downregulation of β-adrenergic receptors [12, 30]. Four of the included studies in this systematic review, however, found that premorbid β-blocker exposure was not associated with a significant difference in vasopressor requirements during sepsis. Similarly, Fuchs et al. found that continuing chronic beta blockers during acute phase of sepsis is not associated with increased use of catecholamines.

Interestingly, Singer et al. reported that patients with premorbid exposure to non-selective β-blockers had lower mortality rates compared to patients with premorbid cardioselective β-blocker exposure [11]. This suggests that β-blocker modulation of non-cardiac adrenergic responses to sepsis may also have an important role. Furthermore, β-blockers may potentially positively modulate the disturbed autonomic (sympathetic-parasympathetic) balance in sepsis [31].

Adrenergic response to sepsis induces a hypermetabolic state characterized by increased energy expenditure, hyperglycaemia, lipolysis and proteolysis, supressed ketogenesis, and negative nitrogen balance resulting in eventual loss of lean body mass [32]. β2-adrenergic blockade appears to have the potential to reverse hyperglycaemia and reduce proteolysis [7]. For example, the use of propranolol in children with severe burns appears to attenuate hypermetabolism and reverse muscle catabolism [33].

The immune system is also modulated by the adrenergic responses to sepsis [34]. The β-adrenergic system regulates apoptosis, mitochondrial function, and inflammatory cytokine production. β-blockers influence the pattern of cytokine synthesis with β1 blockers downregulating a proinflammatory response, whereas β2-antagonization seems to have an opposite effect, at least in chronic heart failure [35].

In sepsis, β2-adrenergic stimulation selectively inhibits CD4^+^ lymphocyte Th1 function and favours the Th2 responses that inhibit macrophage activation, T cell proliferation, and proinflammatory cytokine production [7]. CD8^+^ lymphocyte function may also be suppressed by β2-adrenergic stimulation [36]. The derangement in lymphocytic function induced by catecholamines is thus reminiscent of sepsis-induced immune suppression and could even be considered as one of the mechanisms. However, to date, the evidence for any beneficial use of β-adrenergic blockade on immune function in sepsis has been conflicting [7].

Sepsis results in a pro-thrombotic state with increases in plasma tissue factor and von Willebrand factor levels [37]. Platelets also express adrenergic receptors on their surface [38]. However, there are conflicting effects of β1 and β2 pathways on platelet function [7]. The use of β-adrenergic blockade led to decreased endothelial cell damage in a murine model of shock coagulopathy [39]. This suggests that premorbid β-blocker therapy might mitigate shock-induced endotheliopathy (SHINE), attenuating sepsis-associated coagulopathy [40].

Nonetheless, multiple questions on the role of β-adrenergic blockade in sepsis remain unanswered. On top of safety and efficacy concerns, the duration and dosage at which β-blockade should be performed remain to be elucidated. Furthermore, the timing of therapeutic β-adrenergic blockade initiation is also controversial. The results of our systematic review suggest that we should not discount β-blockers during sepsis. Instead, we may consider continuing chronic β-blockers and perhaps introduce β-blocking drugs early in the sepsis management, especially the non-cardioselective ones.

### Strengths and limitations

This study analysed data from nine observational studies, four of which were reported as conference abstracts. There was not enough data to conduct a meta-analysis. By nature of observational studies, systematic confounding and risk of bias cannot be ruled out. The risk of bias can be reduced by adjusted analysis. Analysis of pooled adjusted odds ratio revealed a significant decrease in sepsis mortality with premorbid β-blocker exposure, but adjusted data were available only from three studies. Despite the three studies providing data on the majority of patients included in this review, substantial heterogeneity is present and residual confounding is likely. Potential sources of confounding include the variable definitions of premorbid β-blocker exposure used by the included studies, the appropriate prescription of β-blockers to all included patients, and patient compliance to treatment.

The conclusions that can be drawn from this study are also hampered by the lack of clinical parameter data, limiting our ability to decipher the likely mechanism/s by which premorbid β-blocker exposure may lower sepsis mortality.

## Conclusion

This systematic review suggests that β-blocker exposure prior to an episode of sepsis could have a role in reducing sepsis mortality. More evidence, however, is needed to elucidate whether premorbid β-blocker treatment is able to mitigate, and by what mechanism, the potentially detrimental effects of endogenous or exogenous catecholamines in early sepsis. Further appropriately powered and ideally prospective observational studies on premorbid β-blocker exposure will be necessary to generate the required evidence.

## Additional files

Additional file 1: **Table S1** MOOSE Checklist. (DOCX 16 kb)

Additional file 2: **Table S2** List of studies excluded from systematic review. (DOCX 14 kb)

Additional file 3: **Table S3** Reasoning for Bias Assessment for Mortality Outcome using ROBINS-1 Tool (DOCX 18 kb)

Additional file 4: **Figure S1** Detailed search strategy. (DOCX 13 kb)

Additional file 5: **Figure S2** Risk of bias assessment for mortality in individual studies using ROBINS-I assessment tool. (TIF 1123 kb)

## Data Availability

Not applicable.

## Abbreviations

aOR: Adjusted odds ratio
APACHE: Acute Physiology and Chronic Health Evaluation
CI: Confidence interval
ED: Emergency department
HR: Hazard ratio
ICU: Intensive care unit
MOOSE: Meta-analysis Of Observational Studies in Epidemiology
PICOS: Patient, Population, or Problem, Intervention, Comparison, Outcome, Study Design or Setting
PROSPERO: International prospective register of systematic reviews
ROBINS-I: Risk Of Bias In Non-randomized Studies - of Interventions
RR: Relative risk
SHINE: Shock-induced endotheliopathy
SOFA score: Sequential Organ Failure Assessment
