# Correction to: The association between premorbid beta blocker exposure and mortality in sepsis—a systematic review

**DOI:** 10.1186/s13054-019-2699-8

**Published:** 2020-01-03

**Authors:** Kaiquan Tan, Martin Harazim, Benjamin Tang, Anthony Mclean, Marek Nalos

**Affiliations:** 1 Nepean Clinical School, Sydney Medical School, University of Sydney, Penrith, Australia; 2 Department of Intensive Care Medicine, Nepean Hospital, Penrith, Australia; 3 Centre for Immunology and Allergy Research, Westmead Millennium Institute, Westmead, Australia; 4 Medical Intensive Care Unit, Teaching Hospital and Biomedical Centre, Charles University, Alej Svobody 80, 323 00 Pilsen, Czech Republic

**Correction to: Crit Care**

**https://doi.org/10.1186/s13054-019-2562-y**

In the publication of this article [1], there was an error in the cited reference 23 [2] within the Family Name. This has now been updated in the original article.

The error: Charles et al.

Should instead read: de Roquetaillade et al.
