# Supplementary material for: The association between premorbid beta blocker exposure and mortality in sepsis—a systematic review
Source: Crit Care. 2019 Sep 4;23:298. doi: 10.1186/s13054-019-2562-y (PMC6727531; doi:10.1186/s13054-019-2562-y)
Supplement: Supplementary file 2 — Table S2 List of studies excluded from systematic review. (DOCX 14 kb) [file 13054_2019_2562_MOESM2_ESM.docx]

| **First author** | **Study title** | **Journal** | **Reason for Exclusion** |
| --- | --- | --- | --- |
| Cliche et al. | Effect of β-blockers and calcium channel blockers on shock index predictability in patients suffering from urosepsis | Critical Care 2013, 17(Suppl 2):P225 (doi: 10.1186/cc12163) | Conference abstract comparing beta blocker and calcium channel blocker use in urosepsis. Beta blocker and calcium channel usage is not separated into two groups. Analysis not possible. |
| Al Harbi et al. | Association between β-blocker use and mortality in critically ill patients: a nested cohort study | Al Harbi et al. BMC Pharmacology and Toxicology (2018) 19:22 https://doi.org/10.1186/s40360-018-0213-6 | Beta blocker usage continued during ICU admission for sepsis episode. Study population included patients with conditions other than sepsis. Adjusted mortality odds ratios was not limited to sepsis population. |
| DeMott et al. | Effects of Chronic Antihypertensives on Vasopressor Dosing in Septic Shock | Annals of Pharmacotherapy 2018, Vol. 52(1) 40–47 | Study cannot be used due to critical risk of bias in selection of participants into study. Study excluded a substantial number of patients on vasopressors prior to MICU admission and restricted vasopressor usage to more than 3 hours. |
| Lee et al. | Preadmission Use of Calcium Channel Blocking Agents Is Associated with Improved Outcomes in Patients with Sepsis: A Population-Based Propensity Score–Matched Cohort Study | Critical Care Medicine 45(9) 2017 | Beta blocker population is only used as an active comparator. Analysis for bias will be for premorbid calcium channel blocker use and mortality which is different from premorbid Beta blocker use and mortality. |
| Maier et al. | Influence of beta-blocker therapy on the risk of infections and death in patients at high risk for stroke induced immunodepression | PLoS ONE 13 (4): e0196174. https://doi.org/10.1371/journal. pone.0196174 | Beta blocker positive group was defined as patients who continued beta blocker therapy during hospital stay. |
| ter Avest et al. | Outcome predictors of uncomplicated sepsis | ter Avest et al. International Journal of Emergency Medicine 2013, 6:9 http://www.intjem.com/content/6/1/9 | Study population was defined as "sepsis in the absence of signs of organ dysfunction at a site remote from the site of the infection" or sepsis without signs of hypotension or hypoperfusion. This is inconsistent with the latest Sepsis-3 definitions of sepsis and septic shock. |
| Wiewel et al. | Prior Use of Calcium Channel Blockers Is Associated with Decreased Mortality in Critically Ill Patients with Sepsis: A Prospective Observational Study | Crit Care Med 2017; 45:454–463 | Calcium channel blockers was the drug under investigation. Information on Beta blocker usage was also presented. Analysis for bias will be for premorbid calcium channel blocker use and mortality, which is different from premorbid Beta blocker use and mortality. |

**Table S2** List of studies excluded from systematic review
