# Supplementary material for: The association between premorbid beta blocker exposure and mortality in sepsis—a systematic review
Source: Crit Care. 2019 Sep 4;23:298. doi: 10.1186/s13054-019-2562-y (PMC6727531; doi:10.1186/s13054-019-2562-y)
Supplement: Supplementary file 3 — Table S3 Reasoning for Bias Assessment for Mortality Outcome using ROBINS-1 Tool (DOCX 18 kb) [file 13054_2019_2562_MOESM3_ESM.docx]

|  | **Bias due to confounding** | | **Bias due to selection of participants into study** | | **Bias in classification of interventions** | | **Bias due to deviations from intended interventions** | | **Bias due to missing data** | | **Bias in measurement of outcomes** | | **Bias in selection of reported result** | |
| --- | --- | --- | --- | --- | --- | --- | --- | --- | --- | --- | --- | --- | --- | --- |
| **First author** | **Risk level** | **Reason** | **Risk level** | **Reason** | **Risk level** | **Reason** | **Risk level** | **Reason** | **Risk level** | **Reason** | **Risk level** | **Reason** | **Risk level** | **Reason** |
| Singer et al. | Moderate | Confounders expected, univariate tests and multivariable logistic regression was performed to correct for confounders | Moderate | Participants were selected by age, but results were adjusted for age via multivariate regression | Low | Patients with beta-blocker prescriptions extending into 30 days prior to admission but not through the admission date were excluded | Low | No systemic differences in treatment between intervention groups | Low | There was no loss to follow up | Low | Measurement of outcome was consistent across intervention groups and unlikely to be influenced by knowledge of intervention received | Moderate | No preregistered protocol or statistical plan available |
| Macchia et al. | Moderate | Confounders expected, univariate tests and multivariable logistic regression was performed to correct for confounders | Moderate | Participants were selected by age, but results were adjusted for age via multivariate regression | Low | Intervention was well defined. | Low | No systemic differences in treatment between intervention groups | Low | There was no loss to follow up | Low | Measurement of outcome was consistent across intervention groups and unlikely to be influenced by knowledge of intervention received | Moderate | No preregistered protocol or statistical plan available |
| Hsieh et al. | Moderate | Confounders expected. Frequency matching, multivariate logistic regression, and stratification analysis was performed to correct for confounders | Low | All eligible participants were included in the study, final participants were derived using frequency matching | Low | Intervention was well defined. | Low | No systemic differences in treatment between intervention groups | Low | There was no to follow up | Low | Measurement of outcome was consistent across intervention groups and unlikely to be influenced by knowledge of intervention received | Moderate | No preregistered protocol or statistical plan available |

**Table S3: Reasoning for Bias Assessment for Mortality Outcome using ROBINS-1 Tool**

|  | **Bias due to confounding** | | **Bias due to selection of participants into study** | | **Bias in classification of interventions** | | **Bias due to deviations from intended interventions** | | **Bias due to missing data** | | **Bias in measurement of outcomes** | | **Bias in selection of reported result** | |
| --- | --- | --- | --- | --- | --- | --- | --- | --- | --- | --- | --- | --- | --- | --- |
| **First author** | **Risk level** | **Reason** | **Risk level** | **Reason** | **Risk level** | **Reason** | **Risk level** | **Reason** | **Risk level** | **Reason** | **Risk level** | **Reason** | **Risk level** | **Reason** |
| Fuchs et al. | Moderate | Confounders expected, univariate tests and multivariable logistic regression was performed to correct for confounders | Low | All eligible participants were included in the study | Low | Intervention was well defined. | Low | No systemic differences in treatment between intervention groups | Low | There was no to follow up | Low | Measurement of outcome was consistent across intervention groups and unlikely to be influenced by knowledge of intervention received | Moderate | No preregistered protocol or statistical plan available |
| Contenti et al. | Serious | Confounders expected, no statistical analysis was performed to correct for confounders for beta blocker usage and mortality outcome | Moderate | Blood lactate levels >2mmol/L was used as a selection criterion for study population | Low | Intervention was well defined. | Low | No systemic differences in treatment between intervention groups | Low | There was no to follow up | Low | Measurement of outcome was consistent across intervention groups and unlikely to be influenced by knowledge of intervention received | Moderate | No preregistered protocol or statistical plan available |

**Table S3 continued: Reasoning for Bias Assessment for Mortality Outcome using ROBINS-1 Tool**

|  | **Bias due to confounding** | | **Bias due to selection of participants into study** | | **Bias in classification of interventions** | | **Bias due to deviations from intended interventions** | | **Bias due to missing data** | | **Bias in measurement of outcomes** | | **Bias in selection of reported result** | |
| --- | --- | --- | --- | --- | --- | --- | --- | --- | --- | --- | --- | --- | --- | --- |
| **First author** | **Risk level** | **Reason** | **Risk level** | **Reason** | **Risk level** | **Reason** | **Risk level** | **Reason** | **Risk level** | **Reason** | **Risk level** | **Reason** | **Risk level** | **Reason** |
| Sharma et al. | Serious | Confounders expected, no statistical analysis was performed to correct for confounders for beta blocker usage and mortality outcome | Low | All eligible participants were included in the study | No Information | No explanation for source of information on how beta blocker exposure was defined | No information | No information is reported on whether there is deviation from the intended intervention. | Low | There was no to follow up | Low | Measurement of outcome was consistent across intervention groups and unlikely to be influenced by knowledge of intervention received | No information | Only abstract available |
| Charles et al. | Serious | Confounders expected, no statistical analysis was performed to correct for confounders for beta blocker usage and mortality outcome | Low | All eligible participants were included in the study | No Information | No explanation for source of information on how beta blocker exposure was defined | No information | No information is reported on whether there is deviation from the intended intervention. | Low | There was no to follow up | Low | Measurement of outcome was consistent across intervention groups and unlikely to be influenced by knowledge of intervention received | No information | Only abstract available |
| Alsolamy et al. | Serious | Confounders expected, no statistical analysis was performed to correct for confounders for beta blocker usage and mortality outcome | Low | All eligible participants were included in the study | No Information | No explanation for source of information on how beta blocker exposure was defined | No information | No information is reported on whether there is deviation from the intended intervention. | Low | There was no to follow up | Low | Measurement of outcome was consistent across intervention groups and unlikely to be influenced by knowledge of intervention received | No information | Only abstract available |
| Al-Qadi et al. | Moderate | Confounders expected, univariate tests and multivariable logistic regression was performed to correct for confounders | Low | All eligible participants were included in the study | Low | Intervention was well defined. | No information | No information is reported on whether there is deviation from the intended intervention. | Low | There was no to follow up | Low | Measurement of outcome was consistent across intervention groups and unlikely to be influenced by knowledge of intervention received | No information | Only abstract available |

**Table S3 continued: Reasoning for Bias Assessment for Mortality Outcome using ROBINS-1 Tool**
