# Supplementary material for: The association between premorbid beta blocker exposure and mortality in sepsis—a systematic review
Source: Crit Care. 2019 Sep 4;23:298. doi: 10.1186/s13054-019-2562-y (PMC6727531; doi:10.1186/s13054-019-2562-y)
Supplement: Supplementary file 4 — Figure S1 Detailed search strategy. (DOCX 13 kb) [file 13054_2019_2562_MOESM4_ESM.docx]

Database: AMED (Allied and Complementary Medicine) <1985 to June 2019>, EBM Reviews - Cochrane Central Register of Controlled Trials <May 2019>, EBM Reviews - Cochrane Clinical Answers <June 2019>, EBM Reviews - Cochrane Database of Systematic Reviews <2005 to June 19, 2019>, EBM Reviews - Cochrane Methodology Register <3rd Quarter 2012>, Embase Classic+Embase <1947 to 2019 June 25>

Search Strategy:

--------------------------------------------------------------------------------

1 adrenergic beta antagonist.mp. or exp adrenergic beta antagonist

2 beta blockers.mp. or exp beta blockers/

3 beta antagonist.mp. or exp beta antagonist/

4 beta adrenoreceptor antagonist.mp. or exp beta adrenoreceptor antagonist/

5 beta adrenergic receptor antagonist.mp. or exp beta adrenergic receptor antagonist/

6 beta adrenergic blocking agent.mp. or exp beta adrenergic blocking agent/

7 adrenergic beta-1 receptor antagonists.mp. or exp Adrenergic beta-1 Receptor Antagonists/

8 acebutolol.mp. or exp Acebutolol/

9 alprenolol.mp. or exp Alprenolol/

10 atenolol.mp. or exp Atenolol/

11 betaxolol.mp. or exp Betaxolol/

12 bisoprolol.mp. or exp Bisoprolol/

13 bunolol.mp. or exp bunolol/

14 bupranolol.mp. or exp Bupranolol/

15 Bucindolol.mp. or exp Bucindolol/

16 carteolol.mp. or exp Carteolol/

17 celiprolol.mp. or exp Celiprolol/

18 Carvedilol.mp. or exp Carvedilol/

19 dihydroalprenolol.mp. or exp Dihydroalprenolol/

20 esmolol.mp. or exp esmolol/

21 iodocyanopindolol.mp. or exp Iodocyanopindolol/

22 labetalol.mp. or exp Labetalol/

23 levobunolol.mp. or exp Levobunolol/

24 metipranolol.mp. or exp Metipranolol/

25 metoprolol.mp. or exp Metoprolol/

26 nadolol.mp. or exp Nadolol/

27 Nebivolol.mp. or exp Nebivolol/

28 oxprenolol.mp. or exp Oxprenolol/

29 penbutolol.mp. or exp Penbutolol/

30 practolol.mp. or exp Practolol/

31 penbutolol.mp. or exp Penbutolol/

32 pindolol.mp. or exp Pindolol/

33 propranolol.mp. or exp Propranolol/

34 sotalol.mp. or exp Sotalol/

35 timolol.mp. or exp Timolol/

36 1 or 2 or 3 or 4 or 5 or 6 or 7 or 8 or 9 or 10 or 11 or 12 or 13 or 14 or 15 or 16 or 17 or 18 or 19 or 20 or 21 or 22 or 23 or 24 or 25 or 26 or 27 or 28 or 29 or 30 or 31 or 32 or 33 or 34 or 35

37 sepsis.mp. or exp Sepsis/

38 septic shock.mp. or exp septic shock/

39 severe sepsis.mp. or exp severe sepsis/

40 septicemia.mp. or exp septicemia/ or septicaemi*.mp. or septicemi*.mp. [mp=ab, hw, ti, ot, sh, kw, tx, ct, tn, dm, mf, dv, fx, dq]

41 bacteraemia.mp. or exp bacteraemia/ or bacteraemi*.mp. or bacteremi*.mp. [mp=ab, hw, ti, ot, sh, kw, tx, ct, tn, dm, mf, dv, fx, dq]

42 37 or 38 or 39 or 40 or 41

43 36 and 42

44 limit 43 to human [Limit not valid in AMED,CCTR,CCA,CDSR,CLCMR; records were retained]

45 limit 44 to humans [Limit not valid in AMED,CCTR,CCA,CDSR,CLCMR; records were retained]

46 remove duplicates from 45

**Figure S1** Detailed search strategy
