# Supplementary figures and images for: The association between premorbid beta blocker exposure and mortality in sepsis—a systematic review
Source: Crit Care. 2019 Sep 4;23:298. doi: 10.1186/s13054-019-2562-y (PMC6727531; doi:10.1186/s13054-019-2562-y)

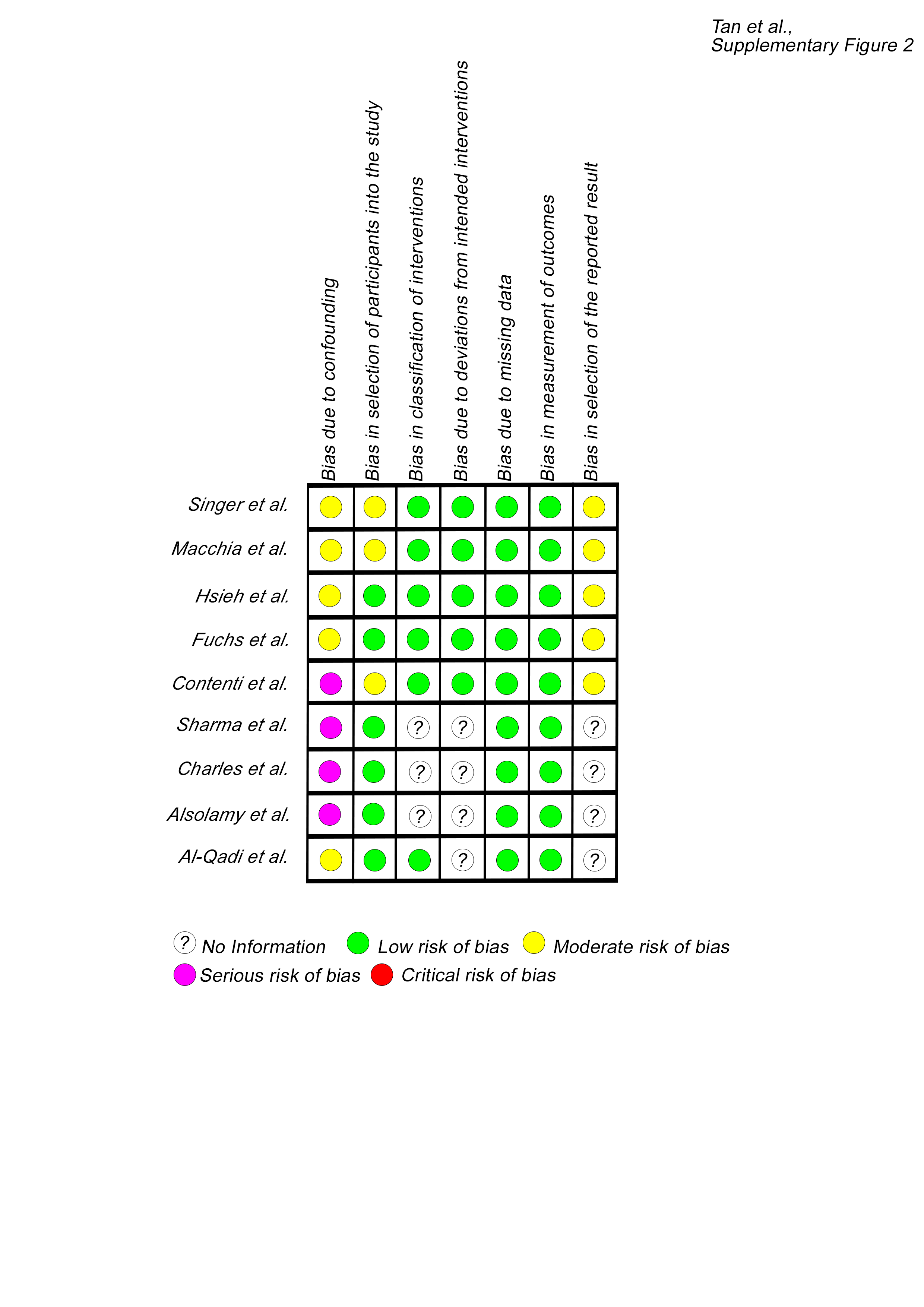

Supplement: Supplementary file 5 — Figure S2 Risk of bias assessment for mortality in individual studies using ROBINS-I assessment tool. (TIF 1123 kb) [file 13054_2019_2562_MOESM5_ESM.tif]
